# Anaerobic Ammonium Oxidation in Acidic Red Soils

**DOI:** 10.3389/fmicb.2018.02142

**Published:** 2018-09-05

**Authors:** Jiapeng Wu, Yiguo Hong, Xiang He, Lijing Jiao, Xiaomei Wen, Shuai Chen, Guangshi Chen, Yiben Li, Tianzheng Huang, Yaohao Hu, Xiaohan Liu

**Affiliations:** ^1^State Key Laboratory of Tropical Oceanography, South China Sea Institute of Oceanology, Chinese Academy of Sciences, Guangzhou, China; ^2^University of Chinese Academy of Sciences, Beijing, China; ^3^School of Environmental Science and Engineering, Guangzhou University, Guangzhou, China

**Keywords:** red soils, anaerobic ammonium oxidation, high-throughput sequencing, *Candidatus* Brocadia, nitrogen removal

## Abstract

Anaerobic ammonium oxidation (anammox) has been proven to be an important nitrogen removal process in terrestrial ecosystems, particularly paddy soils. However, the contribution of anammox in acidic red soils to nitrogen loss has not been well-documented to date. Here, we investigated the activity, abundance, and distribution of anammox bacteria in red soils collected from nine provinces of Southern China. High-throughput sequencing analysis showed that *Candidatus* Brocadia dominates the anammox bacterial community (93.03% of sequence reads). Quantification of the hydrazine synthase gene (*hzsB*) and anammox 16S rRNA gene indicated that the abundance of anammox bacteria ranged from 6.20 × 10^6^ to 1.81 × 10^9^ and 4.81 × 10^6^ to 4.54 × 10^8^ copies per gram of dry weight, respectively. Contributions to nitrogen removal by anammox were measured by a ^15^N isotope-pairing assay. Anammox rates in red soil ranged from 0.01 to 0.59 nmol N g^−1^ h^−1^, contributing 16.67–53.27% to N_2_ production in the studied area, and the total amount of removed nitrogen by anammox was estimated at 2.33 Tg N per year in the natural red soils of southern China. Pearson correlation analyses revealed that the distribution of anammox bacteria significantly correlated with the concentration of nitrate and pH, whereas the abundance and activity of anammox bacteria were significantly influenced by the nitrate and total nitrogen concentrations. Our findings demonstrate that *Candidatus* Brocadia dominates anammox bacterial communities in acidic red soils and plays an important role in nitrogen loss of the red soil in Southern China.

## Introduction

Anaerobic ammonium oxidation (anammox) is a principal microbe-driven process that removes excess N from an ecosystem and oxidizes ${\text{NH}}_{4}^{+}$ with ${\text{NO}}_{2}^{-}$ or ${\text{NO}}_{3}^{-}$ to form N_2_ gas under anaerobic autotrophic condition (Mulder et al., 1995). The discovery of anammox challenged the established concept attributing atmospheric dinitrogen gas (N_2_) from fixed nitrogen in the environment to heterotrophic denitrification. The anammox process is mediated by bacteria belonging to the monophyletic order of *Candidate* Brocadiales, of the phylum *Planctomycetes* (Jetten et al., 2010). Presently, there are five different genera of anammox bacteria that have been described, including *Candidatus* Brocadia (Strous et al., 1999; Kartal et al., 2008), *Candidatus* Jettenia (Quan et al., 2008),*Candidatus* Kuenenia (Schmid et al., 2000), *Candidatus* Anammoxoglobus (Kartal et al., 2007), and *Candidatus* Scalindua (Schmid et al., 2003; Kuypers et al., 2005).

Anammox bacteria are broadly distributed in many kinds of natural environments, including aquatic and terrestrial ecosystems. *Candidatus* Scalindua was almost the only genus found in marine sediments, such as South China Sea (Hong et al., 2011a), Bohai Sea (Dang et al., 2013), and Arabian Sea (Woebken et al., 2008). In contrast to marine environments, all of these genera of anammox bacteria have been detected in soil ecosystems (Wang et al., 2015; Yang et al., 2017). Zhu et al. (2011) found that the 16S rRNA gene sequence of fertilized paddy soil were related to four different genera of anammox bacteria. Anammox bacteria related to *Candidatus* Brocadia, Kuenenia, and two novel unidentified clusters were found to dominate in the 12 typical paddy soils collected in southern China (Yang et al., 2015). Shen et al. (2017) detected three genera of anammox bacteria by Illumina-based 16S rRNA gene sequencing in a vegetable field, including *Candidatus* Kuenenia, Brocadia, and Jettenia. Nonetheless, another study showed that anammox bacteria in rice paddy soils were consisted of mainly *Candidatus* Scalindua (Wang and Gu, 2013), which was regarded as the dominant genus in marine environments. In addition, anammox have been reported to be an important nitrogen removal pathway in soil ecosystems, which accounts for 0.4–37% of the total N_2_ production in soil ecosystems (Zhu et al., 2011; Yang et al., 2015; Shan et al., 2016, 2018; Xi et al., 2016). Yang et al. (2015) suggested that ∼10% of applied N-based fertilizers was lost via the anammox process. Until now, however, there has been limited evidence for the existence and role of anammox bacteria in acidic and natural red soils.

Red soil is widely distributed and covers an area of nearly 2.04 × 10^6^ km^2^ in southern China, accounting for 6.5% of the total farmland area, and is one of China’s most important agricultural soils (Xu et al., 2003). On the other hand, red soils are acidic (pH: 4.2–5.9), nutrient deficient (total N ranges from 1.4 to 2.0 g kg^−1^, Total P: 0.6–0.9 g kg^−1^), poor in organic matter (ranging from 10 to 100 g kg^−1^) and have low water-holding and supplying capacity (Wilson et al., 2004). Fe (total iron: 11.7–148.0 g kg^−1^) and Al oxides are often the dominant clay minerals in red soils. Increasing evidence shows that soil characteristics (e.g., soil moisture, pH, and nutrient conditions) may significantly affect the activity and community of soil ammonia oxidation bacteria and archaea (Nicol et al., 2008; Erguder et al., 2009; Gleeson et al., 2010; Gubryrangin et al., 2011; Liu et al., 2017). We hypothesized that the activity, abundance, and diversity of anammox bacteria in red soil would be lower than in other natural habitats. Besides, the anammox population in red soil is expected to be related to a limited number of genera that can be adapted to acidic and nutrient-deficient conditions.

In this study, 10 representative red soil samples from nine provinces of southern China were collected to (i) analyze the diversity and structure of anammox bacteria by Illumina-based 16S rRNA gene sequencing; (ii) determine the abundance of anammox bacteria by quantitative PCR (qPCR) analysis of the hydrazine synthase gene (*hzsB*) and 16S rRNA gene; (iii) evaluate the potential rates and contribution of anammox and denitrification to N_2_ production using the ^15^N-labeling approach; (iv) analyze the relations between the physicochemical characteristics of red soil and activity, abundance, and community structure of anammox bacteria. This study provides a new understanding of the community composition and N loss contribution of anammox bacteria in non-fertilized acidic red soils.

## Materials and Methods

### Samples Collection of Red Soil

**FIGURE 1:**
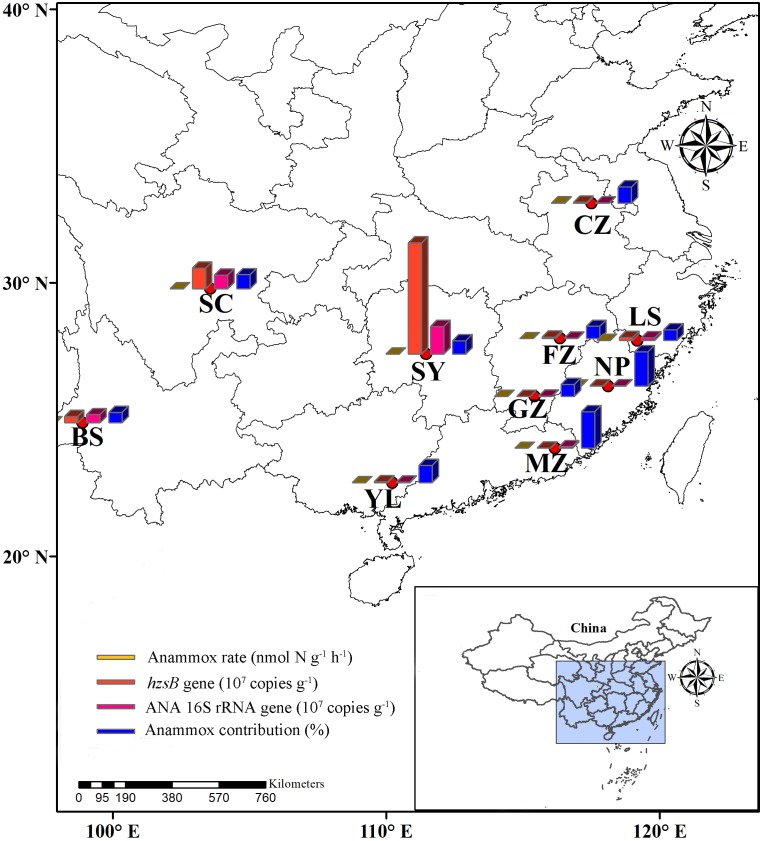
Study area. This figure shows the location of the Southern China and sampling sites of red soils.

A total of 10 red soil samples from nine provinces were collected from the natural fields in southern China (**Figure 1** and **Table 1**) during July 2017. These fields were not affected by a substantial amount of nitrogen fertilizer. Red soil samples (depth: 0–20 cm, **Supplementary Figure 1**) of four replicates (ca. 500 g each subsample) were collected from 10 natural red soils. Soil samples were immediately placed in sterile plastic bags, sealed, and transported to the laboratory on ice. The collected samples were subsequently divided into three parts. The first part was incubated to determine anammox and denitrification activities immediately, the second part was stored at 4°C for subsequent analysis of physicochemical properties, and the third part was stored at −80°C for DNA extraction and molecular analyses.

**Table 1:** Characteristics of the collected red soils in Southern China.

Province	City	Samples	Position	pH	${\text{NH}}_{4}^{+}$ (mg kg^−1^)	${\text{NO}}_{3}^{-}$ (mg kg^−1^)	${\text{NO}}_{2}^{-}$ (mg kg^−1^)	TN (mg kg^−1^)	C (%)	C:N
Yunnan	Baoshan	BS	98°52′ E, 24°53′ N	6.02	69.12	49.17	4.59	570	0.433	7.60
Anhui	Chuzhou	CZ	117°30′ E, 32°54′ N	5.78	29.72	300.61	4.59	120	0.064	5.33
Jiangxi	Fuzhou	FZ	116°20′ E, 27°57′ N	5.18	45.11	14.27	4.77	180	0.116	6.44
Jiangxi	Ganzhou	GZ	115°25′ E, 25°50′ N	4.40	41.23	14.27	3.69	200	0.201	10.05
Zhejiang	Lishui	LS	119°10′ E, 27°53′ N	4.55	42.26	63.56	3.33	130	0.178	13.69
Guangdong	Meizhou	MZ	116°10′ E, 23°57′ N	4.82	39.79	5.49	5.40	70	0.062	8.86
Fujian	Nanping	NP	118°45′ E, 27°32 ′N	5.08	38.80	28.55	2.25	120	0.115	9.58
Sichuan	Leshan	SC	103°32′ E, 29°47′ N	5.06	27.25	3.29	2.25	70	0.056	8.00
Hunan	Shaoyang	SY	111°27′ E, 27°23′ N	4.65	45.18	25.50	5.22	570	0.366	6.42
Guangxi	Yulin	YL	110°12′ E, 22°41′ N	4.56	65.25	10.37	2.70	170	0.238	14.00

### Analyses of Red Soil Physicochemical Properties

pH of red soil was measured at a soil/Milli-Q water ratio of 1:2.5 with a pH analyzer (Mettler Toledo S220, Switzerland). Dissolved inorganic nitrogen (${\text{NH}}_{4}^{+}$, ${\text{NO}}_{2}^{-}$, and ${\text{NO}}_{3}^{-}$) was extracted from the soil using 2 mol/L KCl at a liquid to solid ratio of 100. The extraction process was as follows. Freeze-dried and sieved 100 mg of a sediment sample was placed in a polyethylene centrifuge tube, and a 2 mol/L KCl solution was added to it. It was agitated on a rotary shaker for 8 h. After that, the sediment was centrifuged for 10 min at 3500 rpm, and the supernatant was used to measure the dissolved inorganic nitrogen concentration. Concentrations of ${\text{NH}}_{4}^{+}$, ${\text{NO}}_{2}^{-}$, and ${\text{NO}}_{3}^{-}$ in the supernatants were determined by spectrometric detection methods described by Wu et al. (2016) and Guan et al. (2017). Total nitrogen (TN), organic carbon (%), and the C:N ratio in each sample were analyzed on an elemental analyzer (IsoPrime 100, Elemental, Germany) after leaching with 0.1 M HCl to remove sedimentary carbonate.

### Measurement of Potential Anammox and Denitrification Rates

The potential rates of anammox and denitrification in the red soils were measured as described by Risgaard-Petersen et al. (2003) and Hou et al. (2012) with a slight modification, and their relative contributions to N_2_ production were calculated based on these rates. Briefly, 30 g of field moist soils was transferred to a 250 mL glass bottle with helium-purged water at a volume ratio of 1:5. The mixture was purged with helium for 30 min. The resulting soil slurries were transferred into gas-tight 12.5 mL helium-flushed glass vials (Labco Exetainters, United Kingdom) under helium. Next, these slurries were incubated for approximately 24 h to remove residual ${\text{NO}}_{\text{x}}^{-}$ and dissolved oxygen at *in situ* sampling temperature. The ^15^N atom% (Fn, represents the fraction of ^15^N in total ${\text{NO}}_{3}^{-}$) was calculated by taking into account ^15^N atom% of stock solutions and any residual ambient ${}^{14}{\text{NO}}_{3}^{-}$ as determined by difference, ranging from 0.80 to 0.98. After pre-incubation, these vials with slurries were divided into three groups, which were spiked through the septum with helium-purged stock solutions of (1) ${}^{15}{\text{NH}}_{4}^{+}$ (^15^N at 99.6%), (2) ${}^{15}{\text{NH}}_{4}^{+}{+}^{14}{\text{NO}}_{3}^{-}$, and (3) ${}^{15}{\text{NO}}_{3}^{-}$ (^15^N at 99%). The final concentration of ^15^N in each vial was ∼100 μM. Samples for dissolved gas analysis were preserved with 200 μL of a 50% ZnCl_2_ solution and analyzed within 8 h. Membrane inlet mass spectrometry (MIMS, Hiden) was employed to measure the concentrations of ^29^N_2_ and ^30^N_2_ produced during the incubation period. Finally, the developed methods were used to calculate the rates of both anammox and denitrification and their potential contribution to N_2_ production.

### DNA Extraction and PCR Amplification

Approximately 0.3 g of red soil was used for genomic DNA extraction using the PowerSoil DNA Isolation Kit (Mobio, United States). DNA quality and quantity were checked on a NanoDrop spectrophotometer (NanoDrop Technologies, Wilmington, DE, United States). Anammox bacteria were targeted by the barcoding primers A438f and A684r. The forward primer of A438f was attached to a unique 8 bp barcode sequence. More details of the primers and PCR amplification program are given in **Supplementary Table 1**. The amplified products were verified by electrophoresis in a 1.0% agarose gel and then purified using a MiniBEST agarose gel DNA extraction kit (TaKaRa, Beijing, China) before high-throughput sequencing.

### High-Throughput Sequencing and Analysis

Data analysis of high-throughput raw sequences was conducted in the Mothur software v.1.35.1 following the standard protocol^1^ (Schloss et al., 2009). The obtained reads were processed by removing tags and primers before the sequence read numbers per sample were grouped together. The quality-trimmed sequences were aligned to the newly developed anammox 16S rRNA gene databases. The chimeric sequences were identified and removed by the Chimera-uchime. High-quality anammox bacterial sequences were used to generate a distance matrix and cluster with the average neighbor algorithm. Representative sequences for each operational taxonomic unit (OTU) as defined by 97% sequence identity were obtained for further diversity analyses (Zhao et al., 2013). Phylogenetic analysis of the representative anammox bacterial 16S rRNA gene sequences from each OTU was conducted with the MEGA 7.0 software. A heat map was constructed based on the abundance of top 30 OTUs. Principal coordinates analysis (PCoA) was conducted by Normalized weighted Unifrac (Lozupone et al., 2006). Canonical correspondence analysis (CCA) was performed using the CANOCO 5.0 software. The plots in this study were created in SigmaPlot (version 12.5).

### qPCR Analysis of *hzsB* and 16S rRNA Genes of Anammox Bacteria

The abundance of the *hzsB* gene and 16S rRNA gene of anammox bacteria was determined in triplicate on a Bio-Rad iQ5 thermal cycler (Bio-Rad Laboratories) with primer sets HSBeta396F-HSBeta742R and A438f-A684r, respectively. More details of qPCR assays are given in **Supplementary Table 1**. Plasmids carrying the targeted gene fragments were extracted from *Escherichia coli* DH5α hosts using a Plasmid Mini Preparation Kit (TaKaRa, Beijing, China). Standard curves were built using 10-fold serial dilution of the plasmid with target anammox bacterial genes: *hzsB* and 16S rRNA gene. Specificity of the amplified products was checked by examination of a single melting peak and the presence of a unique band of the expected size in a 2% agarose gel stained with ethidium bromide. The results with efficiency and correlation coefficient above 90% and 0.97 were employed in this study.

### Statistical Analysis

The relations among the activity, abundance of anammox bacteria, and different environmental factors were examined by Pearson correlation analyses using Statistical Analysis System (SAS 9.4).

### Nucleotide Sequence Accession Numbers

The raw Illumina reads of 16S rRNA gene sequences of anammox bacteria were deposited in the NCBI short-read archive under the Accession No. SRP140525.

## Results

### Physicochemical Properties of Red Soils

The concentrations of ${\text{NH}}_{4}^{+}$, ${\text{NO}}_{3}^{-}$, ${\text{NO}}_{2}^{-}$, TN, and C (%) and the ratio C:N of red soils from nine provinces of Southern China are listed in **Table 1**. The pH values of all collected red soil samples were relatively low, ranging from 4.40 to 6.02. High concentrations of ${\text{NH}}_{4}^{+}$ were characteristic of all red soils, ranging from 27.25 to 69.12 mg kg^−1^. ${\text{NO}}_{3}^{-}$ concentrations (ranging from 3.29 to 300.61 mg kg^−1^) were lower than those of ${\text{NH}}_{4}^{+}$ but peaked in sample CZ (300.61 mg kg^−1^) in Anhui Province. The concentrations of ${\text{NO}}_{2}^{-}$ were relatively low and ranged from 2.25 to 5.40 mg kg^−1^. The soil TN and C% contents were lower than those in other agricultural soils, which varied from 70 to 570 mg kg^−1^ and 0.056 to 0.433%, respectively. Such differences may be the result of less application of fertilization in natural red soils. Most of the samples had a low C:N ratio (ranging from 5.33 to 10.05). Nonetheless, samples LS and YL showed relatively higher C:N ratios (13.69 and 14.00). The physicochemical properties of red soils in this study were in the same range as other reported values for red soils (Wilson et al., 2004).

### The Potential Rates of Anammox and Contribution to N_2_ Production

**FIGURE 2:**
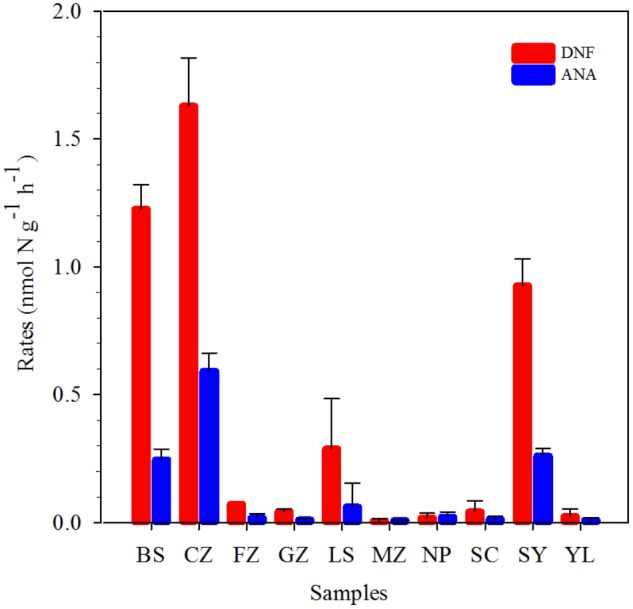
Denitrification (DNF, red) and anammox rates (ANA, blue) in the red soils. Vertical bars denote standard error of triplicate samples.

The potential anammox and denitrification rates were determined in red soils based on the incubation of ${}^{15}{\text{NO}}_{3}^{-}$. The potential anammox rates ranged from 0.01 ± 0.00 to 0.59 ± 0.07 nmol N g^−1^ dry red soil h^−1^, while denitrification rates ranged from 0.01 ± 0.01 to 1.63 ± 0.19 nmol N g^−1^ dry red soil h^−1^ (**Figure 2** and **Supplementary Table 2**). Denitrification rates were higher than anammox rates, suggesting that denitrification dominated the total nitrogen loss in red soils. The highest anammox and denitrification rates were both observed in CZ in Hunan province. The red soil collected in SC, YL, MZ, and GZ showed relatively lower potential anammox rates. The relative contribution of anammox to N_2_ production varied from 16.67 to 53.27%.

### Abundance of Anammox Bacteria

**FIGURE 3:**
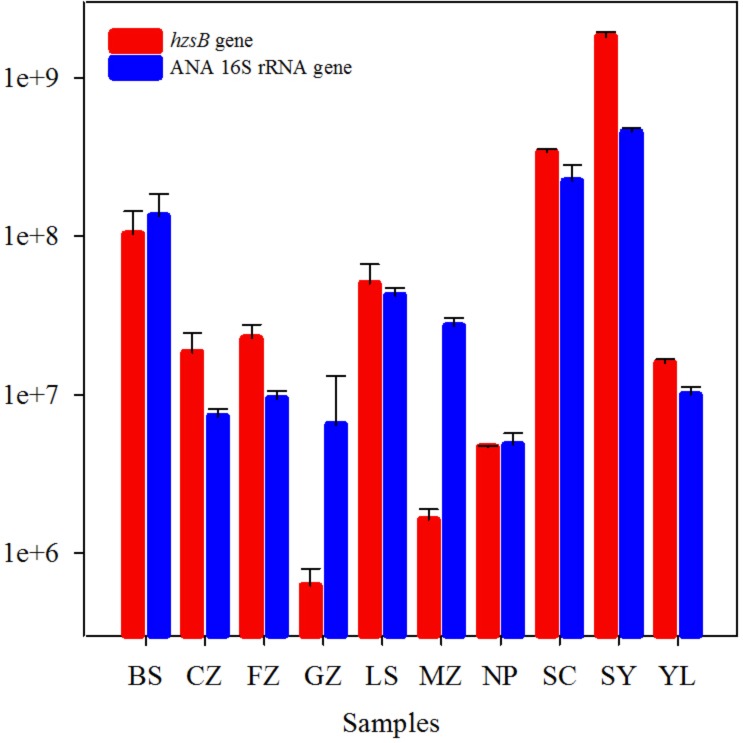
Abundances of hydrazine synthase gene (*hzsB*, red), anammox 16S rRNA gene (blue) in the red soils. Vertical bars denote standard error of triplicate samples.

The *hzsB* gene and anammox 16S rRNA gene were used to estimate the abundance of anammox bacteria in red soils by the qPCR method. The abundance of anammox bacteria ranged from 6.20 × 10^5^ to 1.81 × 10^9^ (*hzsB* gene) and 4.81 × 10^6^ to 4.54 × 10^8^ (anammox 16S rRNA gene) copies per gram of dry weight (**Figure 3**). The highest abundance of anammox bacteria was observed in sample CZ, while other samples had values between 10^5^ and 10^8^ copies/g. *hzsB* was slightly more abundant than the 16S rRNA gene of anammox bacteria (the hzsB/anammox 16S rRNA gene ratio was 2.18) in red soils. The presence of anammox bacteria was ascertained in the current study by the positive correlation between *hzsB* and ANA 16S rRNA gene (*r* = 0.818, *P* < 0.001, *n* = 10).

### Community Composition and Phylogenetic Analysis of Anammox Bacteria

A total of 0.14 million of anammox raw sequences (15000 sequence reads per sample except NP, which contained only 7222 sequences reads) were subjected to denoising and trimming of sequences. After quality controls, ∼12000 reads per sample was filtered as high-quality reads for further analysis (**Table 2**).

**Table 2:** Estimates of sedimentary 16S rRNA gene sequence richness and diversity in different red soils.

Samples	High-quality reads	OTUs^a^	Chao1^a^	ACE^a^	Shannon^a^	Evenness^b^	Coverage
BS	12450	74	89.169	107.027	0.334	0.077	0.996
CZ	11823	77	89.366	130.129	0.542	0.125	0.995
FZ	12425	78	93.469	135.885	0.444	0.102	0.995
GZ	12280	84	105.999	164.916	0.530	0.120	0.995
LS	12501	82	96.499	140.765	0.365	0.083	0.995
MZ	12461	80	95.796	138.615	0.263	0.060	0.995
NP	5824	64	97.833	132.182	0.624	0.150	0.995
SC	12500	81	99.562	148.988	0.320	0.073	0.995
SY	12349	75	91.721	126.715	0.361	0.084	0.996
YL	12404	82	107.521	137.841	0.493	0.112	0.995

*^a^Richness and diversity were determined based on 0.03 distance. ^b^Evenness was calculated by dividing Shannon index by Ln (OTUs).*

Based on the 0.03 distance level, 138 OTUs (77 ± 7 OTUs per sample, *n* = 10) of the anammox 16S RNA gene were obtained (**Table 2**). The highest number of OTUs was seen in sample GZ (84 OTUs). The Chao1 and ACE richness estimators of anammox bacteria ranged from 89.169 to 107.521 and 107.027 to 164.916, respectively. Higher richness of anammox bacteria was found in sample BS. The Shannon index showed that the highest diversity of anammox bacteria was detected in sample NP (0.624), whereas sample MZ (0.263) had the lowest diversity among red soils. The evenness of anammox bacteria was low in red soil and ranged from 0.060 to 0.150. Good’s coverage values varied from 0.995 to 0.996, suggesting that the libraries were adequately large. Rarefaction curves (**Supplementary Figure 2**) and a rank-abundance curve (**Supplementary Figure 3**) for the anammox bacterial 16S rRNA gene at 97% similarity showed that the high-throughput sequencing could supply enough bioinformation to investigate the community composition and diversity of anammox bacteria in the current study.

**FIGURE 4:**
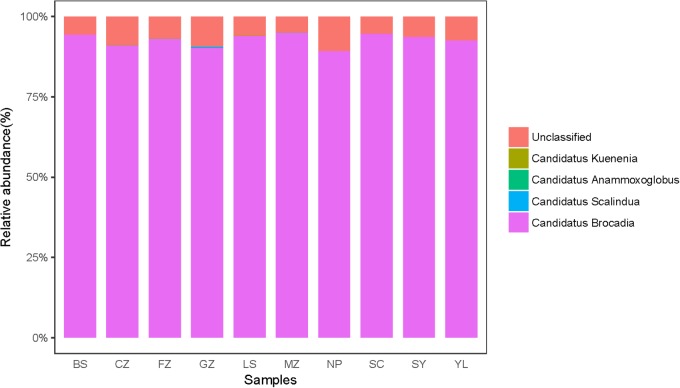
Taxonomic classifications of anammox 16S rRNA gene reads retrieved from the red soils at genus level.

**FIGURE 5:**
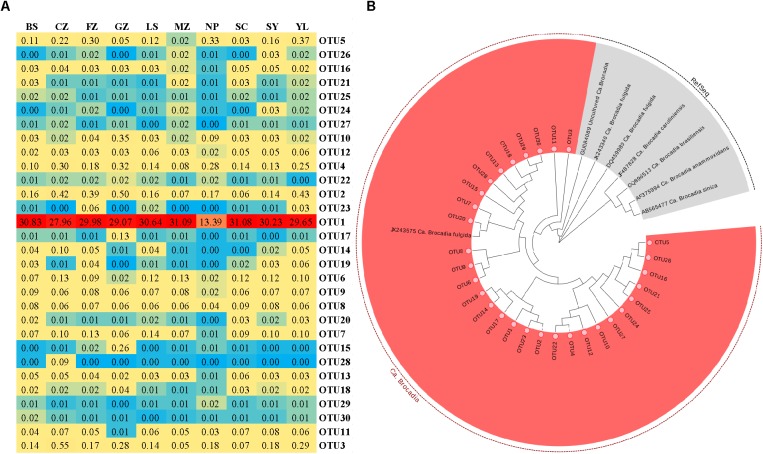
Microbial characteristics of the anammox 16S rRNA gene in the red soils. **(A)** The heat map of the most abundant anammox OTUs (Top 30 OTUs, 97% cutoff). **(B)** Neighbor-joining phylogenetic tree of the dominant (Top 30 OTUs) anammox OTUs and the reference sequences from GenBank. Bootstrap values were 1000 replicates.

The community structure of anammox bacteria in red soil at the genus level is depicted in **Figure 4**. Gene sequences of anammox bacteria mainly belonged to *Candidatus* Brocadia (93.03%), *Candidatus* Scalindua (0.09%), *Candidatus* Anammoxoglobus (0.01%), *Candidatus* Kuenenia (0.04%) and unclassified (6.83%). *Candidatus* Brocadia (93.03%) was the dominant genus in acidic and natural red soils, whereas *Candidatus* Scalindua was more abundant in sample GZ. A heatmap (**Figure 5A**) and phylogenetic tree (**Figure 5B** and **Supplementary Figure 4**) of dominant anammox OTUs (top 30 OTUs, 99% sequences were obtained) indicated that the top 30 OTUs were affiliated with only one cluster, which was closely related to *Candidatus* Brocadia fulgida. OTU 1 was the most abundant sequence in all the samples and had average relative abundance of 27.39.

### Correlation Analysis of Anammox Potential Rates, Gene Abundances, and Red Soil Properties

**FIGURE 6:**
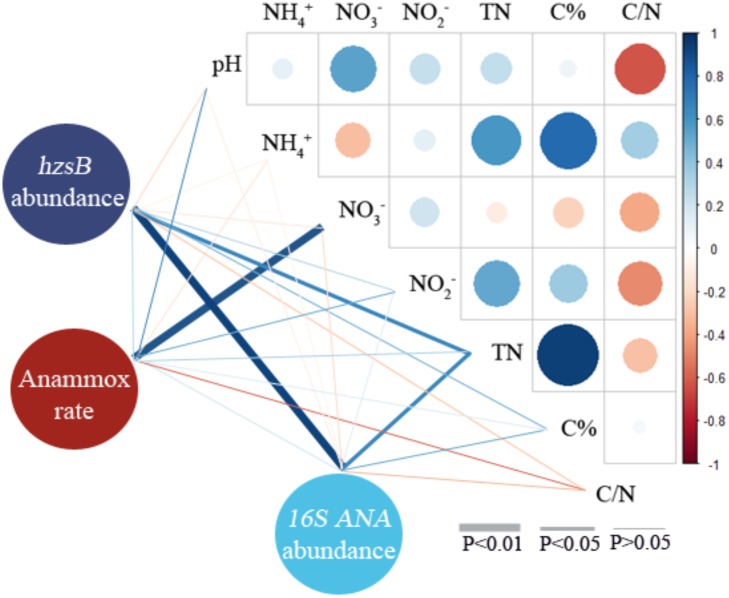
Pearson correlation analyses of anammox rate, *hzsB* abundance, anammox 16S rRNA gene abundance, and environmental characteristics of red soils (including pH, ${\text{NH}}_{4}^{+}$, ${\text{NO}}_{3}^{-}$, ${\text{NO}}_{2}^{-}$, TN, C%, and C/N). Blue and red denote positive and negative correlations, respectively.

Pearson correlation analysis was used to illustrate the correlations among anammox potential rates, gene abundances, and red soil properties (**Figure 6** and **Supplementary Table 3**). The results indicated that anammox rates positively correlated with ${\text{NO}}_{3}^{-}$ (coefficient = 0.889, *P* < 0.001). *hzsB* abundances positively correlated with TN (coefficient = 0.637, *P* < 0.05) and anammox 16S rRNA abundances (coefficient = 0.942, *P* < 0.001). Anammox 16S rRNA abundance also positively correlated with TN (coefficient = 0.652, *P* < 0.05). In addition, C% positively correlated with ${\text{NH}}_{4}^{+}$ (coefficient = 0.779, *P* < 0.001) and TN (coefficient = 0.993, *P* < 0.001) in red soils. As revealed by CCA, pH and ${\text{NO}}_{3}^{-}$ were found to be the main factors affecting the distribution of anammox bacteria in the red soils (**Supplementary Figure 5**).

## Discussion

Recently, increasing evidences showed that anammox bacteria play a significant role in nitrogen loss in agricultural soils, particularly paddy soils (Zhu et al., 2011; Wang and Gu, 2013; Wang et al., 2014; Nie et al., 2015; Yang et al., 2015). In the present study, we present the evidence of anammox process for the first time in 10 different acidic red soils of Southern China by combining the ^15^N-labeling approach, qPCR analysis, and high-throughput sequencing. The potential anammox rates, ranging from 0.01 ± 0.00 to 0.59 ± 0.07 nmol N g^−1^ dry red soil h^−1^ (**Figure 2** and **Supplementary Table 2**), were examined in all the selected red soils, revealing the same range as that found in agricultural soils (Long et al., 2013; Nie et al., 2015) and forest soils (Xi et al., 2016), but lower than other values reported for fertilized paddy soils and agricultural fields (Zhu et al., 2011; Yang et al., 2015; Shen et al., 2016, 2017). Red soils in China were found to be characterized by low pH, TN, and organic carbon, in contrast to other fertilized agricultural soils (**Table 1**). The physicochemical properties of red soils were found to be the most important factors influencing the potential anammox and denitrification rates, especially soil organic carbon and TN. Soil organic carbon can enhance microbial activities and improve physical and chemical conditions of the soil (Pulleman et al., 2000; Zhang et al., 2009). These results indicated that the characteristics of red soil may influence the activity of anammox bacteria. Other studies also showed that flooding and fertilization conditions made the paddy soils a suitable habitat for anammox bacteria (Shen et al., 2017). Zhu et al. (2011) found many microniches were existed in a paddy soil; thus, it could support the various ecophysiologies of the different anammox genera and facilitate the anammox activity. In addition, it should be noticed that slurry incubations may underestimate the *in situ* anammox activity due to the following reasons. Firstly, the incubation conditions at higher moisture may affect the microbial community, which results in underestimation of the denitrification and anammox rate. Finally, the incubation time of 24 h may not be enough to eliminate the initial ${}^{14}{\text{NO}}_{3}^{-}$ in sample CZ (Fn: 0.80) and LS (Fn: 0.87), which may underestimate the potential rates. Different potential rates of anammox were observed in 10 samples from nine provinces of Southern China (**Figure 2**). Cultivation history, land use patterns, and a cropping system are the important factors influencing the physicochemical properties of red soil in different provinces (Shen et al., 2013). Anammox contributed 16.67 to 53.27% to red soil N_2_ production (**Figure 2** and **Supplementary Table 2**), suggesting that anammox should be recognized as a significant N sink in red soil. On the base of the soil density, the average potential anammox rate, and red soil area in southern China, N loss attributed to anammox was estimated to reach∼ 2.33 Tg N per year. This result was comparable to previously reported values (2.50 Tg N per year) in paddy soil in Southern China (Yang et al., 2015), demonstrating the importance of anammox in red soil ecosystems. Pearson correlation analyses showed that the potential anammox rates positively correlated with ${\text{NO}}_{3}^{-}$ (coefficient = 0.889, *P* < 0.001) (**Figure 6** and **Supplementary Table 3**). Previous study on freshwater sediments also suggested that ${\text{NO}}_{3}^{-}$ is a factor controlling anammox rates (Yoshinaga et al., 2011). Anammox bacteria are favored in environments where ${\text{NO}}_{3}^{-}$ is available (Trimmer et al., 2003; Engström et al., 2005; Yang et al., 2015).

The abundance of anammox bacteria ranged from 6.20 × 10^5^ to 1.81 × 10^9^ *hzsB* copies/g and 4.81 × 10^6^ to 4.54 × 10^8^ (anammox bacterial 16S rRNA gene), which fall within the ranges reported in Pearl River Estuary (1.4–20 × 10^8^ *hzsB* copies/g; Wang et al., 2012), Yangtze Estuary (3.67–822 × 10^5^ 16S rRNA copies/g; Zheng et al., 2016), and paddy soils (0.7–1.4 × 10^7^ *hzs* copies/g; Nie et al., 2015). Anammox bacterial abundances in red soils were confirmed by means of the anammox *hzsB* gene and 16S rRNA gene (**Figure 3**), and abundances of these two target genes correlated (*r* = 0.818, *P* < 0.001, *n* = 10). In this study, TN concentration was identified as an important factor influencing the anammox bacterial abundance in selected red soils (**Figure 6** and **Supplementary Table 3**). It can be speculated that higher TN concentration may provide an environment favorable for the distribution and growth of anammox bacteria (Shen et al., 2016). In line with other studies (Metz et al., 2003; Etchebehere and Tiedje, 2005; Yang et al., 2017), a positive correlation between anammox activity and abundance (*hzsB* or 16S rRNA gene) was not observed. These results indicated that anammox rates are limited not by the presence of the population capable of anammox but by the availability of ${\text{NO}}_{3}^{-}$ or ${\text{NH}}_{4}^{+}$ as substrate. If anammox bacterial abundance correlated with their activities, it suggested that anammox bacterial abundance has a potential to predict anammox activity (Dale et al., 2009). Concentrations of ${\text{NO}}_{3}^{-}$ and/or ${\text{NH}}_{4}^{+}$ have been reported to significantly affect the anammox rate (Shen et al., 2013). Lisa et al. (2015) found that physiological nature of an organism and the environmental conditions of the system are the two important factors influencing whether the genetic potential may or may not predict the potential for that process. Furthermore, the presence of anammox bacteria detected by gene amplification is not an indication of their level of activity as would be indicated by active community (cDNA) qualification.

Some studies have reported that *Candidatus* Scalindua is the exclusive anammox genus in marine ecosystems (Schmid et al., 2007; Hong et al., 2011a,b). In this study, *Candidatus* Brocadia dominated the anammox community in acidic red soils. In total, 93.03% sequence reads of anammox bacteria were related to *Candidatus* Brocadia (**Figure 4**) and the dominant OTUs (TOP 30) were all affiliated with *Candidatus* Brocadia fulgida (**Figure 5**). *Candidatus* Brocadia was reported to be the most common anammox genus in terrestrial ecosystems, including paddy soils (50%, Yang et al., 2015) and Chinese agricultural soils (75%, Shen et al., 2013). Some studies showed that *Candidatus* Brocadia possesses diverse metabolic pathways (Gori et al., 2011) and better adaptability to terrestrial soils than do other anammox genera (Shen et al., 2013). Kartal et al. (2008) found that *Candidatus* Brocadia fulgida can use short-chain organic acids as alternative electron donors to reduce ${\text{NO}}_{2}^{-}$ to N_2_. *Candidatus* Brocadia belongs to the r-strategists, making it suitable for the higher substrate concentration, and it will outcompete other anammox genera (Strous, 1999; Gao et al., 2018). CCA suggests that pH and ${\text{NO}}_{3}^{-}$ were the main factors affecting the distribution of anammox bacteria in the red soils (**Supplementary Figure 5**). According to the composition of anammox bacteria communities of different samples (**Table 2** and **Figure 5A**), higher Shannon (0.542) and evenness indexes (0.125) were detected in sample CZ with higher pH (5.78) and ${\text{NO}}_{3}^{-}$ values (300.61 mg kg^−1^). This situation may lead to the higher diversity of anammox bacteria observed in samples with higher pH and ${\text{NO}}_{3}^{-}$ content. Here, the presence and activity of anammox bacteria were detected successfully in acidic red soils (pH ranged from 4.40 to 6.02). The reported optimal pH range for *Candidatus* Brocadia fulgida is 7.2 to 8.3 (Oshiki et al., 2016). Our study extended the pH range in which anammox bacteria can survive and their activity can be detected. A special membrane probably present in the cell of anammox bacteria, so they can survive in acidic environments. Yang et al. (2015) also found that pH significantly correlates with the anammox bacterial composition in paddy soils. Additionally, ${\text{NO}}_{3}^{-}$ content had a significant contribution to anammox bacterial community structure, which may be attributed to an increased supply of ${\text{NO}}_{2}^{-}$ via reduction of ${\text{NO}}_{3}^{-}$. Similar results have also been observed in the sediments of the Dongjiang River (Sun et al., 2013) and Mai Po Nature Reserve (Li et al., 2011).

Thus, both the presence and activity of anammox bacteria were for the first time shown in 10 different acidic red soils from nine provinces in Southern China. These data expand the knowledge of the distribution and N loss contribution of anammox bacteria in acidic red soils. Our high-throughput sequencing analysis indicates that *Candidatus* Brocadia dominates the anammox bacterial community. Anammox contributed 16.67 to 53.27% to red soil N_2_ production, which was the vital N sink (2.33 Tg N per year) in red soils of Southern China. These results demonstrate the lower diversity but higher contribution of anammox bacteria to the removal of fixed N from acidic red soils.

## Author Contributions

JW, YH, XH, LJ, and XW performed the research. JW, YH, SC, GC, YL, TH, YHH, and XL analyzed the data. JW and YH wrote the paper. All co-authors substantially contributed to commenting and revising it and read and approved the final manuscript.

## Conflict of Interest Statement

The authors declare that the research was conducted in the absence of any commercial or financial relationships that could be construed as a potential conflict of interest.

## Footnotes

1 http://www.mothur.org

## References

[B1] Dale O. R. Tobias C. R. Song B. (2009). Biogeographical distribution of diverse anaerobic ammonium oxidizing (anammox) bacteria in cape fear river estuary. *Environ. Microbiol.* 11 1194–1207. 10.1111/j.1462-2920.2008.01850.x 19161435

[B2] Dang H. Zhou H. Zhang Z. Yu Z. Hua E. Liu X. (2013). Molecular detection of *Candidatus* scalindua pacifica and environmental responses of sediment anammox bacterial community in the bohai sea, China. *PLoS One* 8:e61330. 10.1371/journal.pone.0061330 23577216 PMC3620062

[B3] Engström P. Dalsgaard T. Hulth S. Aller R. C. (2005). Anaerobic ammonium oxidation by nitrite (anammox): implications for N_2_ production in coastal marine sediments. *Geochim. Cosmochim. Acta* 69 2057–2065. 10.1016/j.gca.2004.09.032

[B4] Erguder T. H. Boon N. Wittebolle L. Marzorati M. Verstraete W. (2009). Environmental factors shaping the ecological niches of ammonia-oxidizing archaea. *FEMS Microbiol. Rev.* 33 855–869. 10.1111/j.1574-6976.2009.00179.x 19453522

[B5] Etchebehere C. Tiedje J. (2005). Presence of two different active nirS nitrite reductase genes in a denitrifying *Thauera* sp. from a high-nitrate-removal-rate reactor. *Appl. Environ. Microbiol.* 71 5642–5645. 10.1128/AEM.71.9.5642-5645.2005 16151169 PMC1214690

[B6] Gao D. Wang X. Liang H. Wei Q. Dou Y. Li L. (2018). Anaerobic ammonia oxidizing bacteria: ecological distribution, metabolism, and microbial interactions. *Front. Environ. Sci. Eng.* 12:10 10.1007/s11783-018-1035-x

[B7] Gleeson D. B. Müller C. Banerjee S. Ma W. Siciliano S. D. Murphy D. V. (2010). Response of ammonia oxidizing archaea and bacteria to changing water filled pore space. *Soil Biol. Biochem.* 42 1888–1891. 10.1016/j.soilbio.2010.06.020

[B8] Gori F. Tringe S. G. Kartal B. Marchiori E. Machiori E. Jetten M. S. (2011). The metagenomic basis of anammox metabolism in *Candidatus ‘Brocadia fulgida’*. *Biochem. Soc. Trans.* 39 1799–1804. 10.1042/BST20110707 22103529

[B9] Guan F. Hong Y. Jiapeng W. U. Wang Y. Liying B. Tang B. (2017). A fast sodium hypobromite oxidation method for the sequential determination of ammonia nitrogen in small volume. *Ecol. Sci.* 36 42–48.

[B10] Gubryrangin C. Hai B. Quince C. Engel M. Thomson B. C. James P. (2011). Niche specialization of terrestrial archaeal ammonia oxidizers. *Proc. Natl. Acad. Sci. U.S.A.* 108 21206–21211. 10.1073/pnas.1109000108 22158986 PMC3248517

[B11] Hong Y. G. Li M. Cao H. Gu J. D. (2011a). Residence of Habitat-Specific anammox bacteria in the deep-sea subsurface sediments of the south China sea: analyses of marker gene abundance with physical chemical parameters. *Microb. Ecol.* 62 36–47. 10.1007/s00248-011-9849-0 21491114 PMC3141849

[B12] Hong Y. G. Yin B. Zheng T. L. (2011b). Diversity and abundance of anammox bacterial community in the deep-ocean surface sediment from equatorial Pacific. *Appl. Microbiol. Biotechnol.* 89 1233–1241. 10.1007/s00253-010-2925-4 20949269

[B13] Hou L. Liu M. Carini S. A. Gardner W. S. (2012). Transformation and fate of nitrate near the sediment–water interface of copano bay. *Cont. Shelf Res.* 35 86–94. 10.1016/j.csr.2012.01.004 23706021

[B14] Jetten M. S. M. Op den Camp H. J. M. Kuenen J. G. Strous M. (2010). “Description of the order brocadiales,” in *Bergey’s Manual of Systematic Bacteriology*, Vol. 4 eds Krieg N. R. Whitman W. W. Ludwig and W. B. (Heidelberg: Springer), 596–603.

[B15] Kartal B. Rattray J. Niftrik L. A. V. Vossenberg J. V. D. Schmid M. C. Webb R. I. (2007). *Candidatus* “Anammoxoglobus propionicus” a new propionate oxidizing species of anaerobic ammonium oxidizing bacteria. *Syst. Appl. Microbiol.* 30 39–49. 10.1016/j.syapm.2006.03.004 16644170

[B16] Kartal B. Van Niftrik L. Rattray J. van de Vossenberg J. L. Schmid M. C. Sinninghe D. J. (2008). *Candidatus* ‘*Brocadia fulgida’*: an autofluorescent anaerobic ammonium oxidizing bacterium. *FEMS Microbiol. Ecol.* 63 46–55. 10.1111/j.1574-6941.2007.00408.x 18081590

[B17] Kuypers M. M. Lavik G. Woebken D. Schmid M. Fuchs B. M. Amann R. (2005). Massive nitrogen loss from the benguela upwelling system through anaerobic ammonium oxidation. *Proc. Natl. Acad. Sci. U.S.A.* 102 6478–6483. 10.1073/pnas.0502088102 15843458 PMC556276

[B18] Li M. Cao H. Hong Y. G. Gu J. D. (2011). Seasonal dynamics of anammox bacteria in estuarial sediment of the mai po nature reserve revealed by analyzing the 16S rRNA and hydrazine oxidoreductase (hzo) genes. *Microbes Environ.* 26 15–22. 10.1264/jsme2.ME10131 21487198

[B19] Lisa J. A. Song B. Tobias C. R. Hines D. E. (2015). Genetic and biogeochemical investigation of sedimentary nitrogen cycling communities responding to tidal and seasonal dynamics in cape fear river estuary. *Estuar. Coastal Shelf Sci.* 167 A313–A323. 10.1016/j.ecss.2015.09.008

[B20] Liu H. Wu X. Wang Q. Wang S. Liu D. Liu G. (2017). Responses of soil ammonia oxidation and ammonia-oxidizing communities to land-use conversion and fertilization in an acidic red soil of southern China. *Eur. J. Soil Biol.* 80 110–120. 10.1016/j.ejsobi.2017.05.005

[B21] Long A. Heitman J. Tobias C. Philips R. Song B. (2013). Co-occurring anammox, denitrification, and codenitrification in agricultural soils. *Appl. Environ. Microbiol.* 79 168–176. 10.1128/AEM.02520-12 23087029 PMC3536082

[B22] Lozupone C. Hamady M. Knight R. (2006). UniFrac–an online tool for comparing microbial community diversity in a phylogenetic context. *BMC Bioinformatics* 7:371. 10.1186/1471-2105-7-371 16893466 PMC1564154

[B23] Metz S. Beisker W. Hartmann A. Schloter M. (2003). Detection methods for the expression of the dissimilatory copper-containing nitrite reductase gene (DnirK) in environmental samples. *J. Microbiol. Methods* 55 41–50. 10.1016/S0167-7012(03)00089-7 14499994

[B24] Mulder A. van de Graaf A. A. Robertson L. A. Kuenen J. G. (1995). Anaerobic ammonium oxidation discovered in a denitrifying fluidized bed reactor. *FEMS Microbiol. Ecol.* 16 177–184. 10.1111/j.1574-6941.1995.tb00281.x

[B25] Nicol G. W. Leininger S. Schleper C. Prosser J. I. (2008). The influence of soil pH on the diversity, abundance and transcriptional activity of ammonia oxidizing archaea and bacteria. *Environ. Microbiol.* 10 2966–2978. 10.1111/j.1462-2920.2008.01701.x 18707610

[B26] Nie S. Li H. Yang X. Zhang Z. Weng B. Huang F. (2015). Nitrogen loss by anaerobic oxidation of ammonium in rice rhizosphere. *ISME J.* 9 2059–2067. 10.1038/ismej.2015.25 25689022 PMC4542037

[B27] Oshiki M. Satoh H. Okabe S. (2016). Ecology and physiology of anaerobic ammonium oxidizing bacteria. *Environ. Microbiol.* 18 2784–2796. 10.1111/1462-2920.13134 26616750

[B28] Pulleman M. M. Bouma J. Essen E. A. V. Meijles E. W. (2000). Soil organic matter content as a function of different land use history. *Soil Sci. Soc. Am. J.* 64 689–693. 10.2136/sssaj2000.642689x

[B29] Quan Z. X. Rhee S. K. Zuo J. E. Yang Y. Bae J. W. Park J. R. (2008). Diversity of ammonium-oxidizing bacteria in a granular sludge anaerobic ammonium-oxidizing (anammox) reactor. *Environ. Microbiol.* 10 3130–3139. 10.1111/j.1462-2920.2008.01642.x 18479446

[B30] Risgaard-Petersen N. Nielsen L. P. Rysgaard S. Dalsgaard T. Meyer R. L. (2003). Application of the isotope pairing technique in sediments where anammox and denitrification coexist. *Limnol. Oceanogr. Methods* 1 63–73. 10.4319/lom.2003.1.63

[B31] Schloss P. D. Westcott S. L. Ryabin T. Hall J. R. Hartmann M. Hollister E. B. (2009). Introducing mothur: open-source, platform-independent, community-supported software for describing and comparing microbial communities. *Appl. Environ. Microbiol.* 75 7537–7541. 10.1128/AEM.01541-09 19801464 PMC2786419

[B32] Schmid M. Twachtmann U. Klein M. Strous M. Juretschko S. Jetten M. (2000). Molecular evidence for genus level diversity of bacteria capable of catalyzing anaerobic ammonium oxidation. *Syst. Appl. Microbiol.* 23 93–106. 10.1016/S0723-2020(00)80050-8 10879983

[B33] Schmid M. Walsh K. Webb R. Rijpstra W. I. van de Pas-Schoonen K. Verbruggen M. J. (2003). *Candidatus* “Scalindua brodae”, sp. nov., *Candidatus “Scalindua wagneri”*, sp. nov., two new species of anaerobic ammonium oxidizing bacteria. *Syst. Appl. Microbiol.* 26 529–538. 10.1078/072320203770865837 14666981

[B34] Schmid M. C. Risgaardpetersen N. van de Vossenberg J. Kuypers M. M. Lavik G. Petersen J. (2007). Anaerobic ammonium-oxidizing bacteria in marine environments: widespread occurrence but low diversity. *Environ. Microbiol.* 9 1476–1484. 10.1111/j.1462-2920.2007.01266.x 17504485

[B35] Shan J. Yang P. Shang X. Rahman M. M. Yan X. (2018). Anaerobic ammonium oxidation and denitrification in a paddy soil as affected by temperature, pH, organic carbon, and substrates. *Biol. Fertil. Soils* 54 341–348. 10.1007/s00374-018-1263-z

[B36] Shan J. Zhao X. Sheng R. Xia Y. Ti C. Quan X. (2016). Dissimilatory nitrate reduction processes in typical chinese paddy soils: rates, relative contributions, and influencing factors. *Environ. Sci. Technol.* 50 9972–9980. 10.1021/acs.est.6b01765 27499451

[B37] Shen L. Wu H. Liu X. Li J. (2017). Vertical distribution and activity of anaerobic ammonium-oxidising bacteria in a vegetable field. *Geoderma* 288 56–63. 10.1016/j.geoderma.2016.11.007

[B38] Shen L. Zheng P. Ma S. (2016). Nitrogen loss through anaerobic ammonium oxidation in agricultural drainage ditches. *Biol. Fertil. Soils* 52 127–136. 10.1007/s00374-015-1058-4

[B39] Shen L. D. Liu S. Lou L. P. Liu W. P. Xu X. Y. Zheng P. (2013). Broad distribution of diverse anaerobic ammonium-oxidizing bacteria in chinese agricultural soils. *Appl. Environ. Microbiol.* 79 6167–6172. 10.1128/AEM.00884-13 23747706 PMC3811364

[B40] Strous M. (1999). Key physiology of anaerobic ammonium oxidation. *Appl. Environ. Microbiol.* 65 3248–3250. 10388731 10.1128/aem.65.7.3248-3250.1999 PMC91484

[B41] Strous M. Fuerst J. A. Kramer E. H. Logemann S. Muyzer G. van de Pas-Schoonen K. T. (1999). Missing lithotroph identified as new planctomycete. *Nature* 400 446–449. 10.1038/22749 10440372

[B42] Sun W. Xu M. Wu W. M. Guo J. Xia C. Sun G. (2013). Molecular diversity and distribution of anammox community in sediments of the dongjiang river, a drinking water source of Hong Kong. *J. Appl. Microbiol.* 116 464–476. 10.1111/jam.12367 24125160

[B43] Trimmer M. Nicholls J. C. Deflandre B. (2003). Anaerobic ammonium oxidation measured in sediments along the thames estuary, United Kingdom. *Appl. Environ. Microbiol.* 69 6447–6454. 10.1128/AEM.69.11.6447-6454.2003 14602599 PMC262292

[B44] Wang J. Dong H. Wang W. Gu J. D. (2014). Reverse-transcriptional gene expression of anammox and ammonia-oxidizing archaea and bacteria in soybean and rice paddy soils of northeast China. *Appl. Microbiol. Biotechnol.* 98 2675–2686. 10.1007/s00253-013-5242-x 24077726

[B45] Wang J. Gu J. D. (2013). Dominance of *Candidatus* Scalindua species in anammox community revealed in soils with different duration of rice paddy cultivation in northeast China. *Appl. Microbiol. Biotechnol.* 97 1785–1798. 10.1007/s00253-012-4036-x 22526793 PMC3562551

[B46] Wang S. Hong Y. Wu J. Xu X. R. Bin L. Pan Y. (2015). Comparative analysis of two 16S rRNA gene-based PCR primer sets provides insight into the diversity distribution patterns of anammox bacteria in different environments. *Appl. Microbiol. Biotechnol.* 99 8163–8176. 10.1007/s00253-015-6814-8 26231134

[B47] Wang S. Zhu G. Peng Y. Jetten M. S. M. Yin C. (2012). Anammox bacterial abundance, activity, and contribution in riparian sediments of the pearl river estuary. *Environ. Sci. Technol.* 46 8834–8842. 10.1021/es3017446 22816681

[B48] Wilson M. J. He Z. Yang X. (2004). *The Red Soils of China.* Alphen aan den Rijn: Kluwer Academic Publishers 10.1007/978-1-4020-2138-1

[B49] Woebken D. Lam P. Kuypers M. M. Naqvi S. W. Kartal B. Strous M. (2008). A microdiversity study of anammox bacteria reveals a novel *Candidatus* Scalindua phylotype in marine oxygen minimum zones. *Environ. Microbiol.* 10 3106–3119. 10.1111/j.1462-2920.2008.01640.x 18510553

[B50] Wu J. Hong Y. Guan F. Wang Y. Tan Y. Yue W. (2016). A rapid and high-throughput microplate spectrophotometric method for field measurement of nitrate in seawater and freshwater. *Sci. Rep.* 6:20165. 10.1038/srep20165 26832984 PMC4735594

[B51] Xi D. Bai R. Zhang L. Fang Y. (2016). Contribution of anammox to nitrogen removal in two temperate forest soils. *Appl. Environ. Microbiol.* 82 4602–4612. 10.1128/AEM.00888-16 27208117 PMC4984287

[B52] Xu R. Zhao A. Li Q. Kong X. Ji G. (2003). Acidity regime of the Red Soils in a subtropical region of southern China under field conditions. *Geoderma* 115 75–84. 10.1016/S0016-7061(03)00077-6

[B53] Yang X. R. Li H. Nie S. A. Su J. Q. Weng B. S. Zhu G. B. (2015). Potential contribution of anammox to nitrogen loss from paddy soils in southern China. *Appl. Environ. Microbiol.* 81 938–947. 10.1128/AEM.02664-14 25416768 PMC4292472

[B54] Yang X. R. Weng B. S. Li H. Marshall C. W. Li H. Chen Y. S. (2017). An overlooked nitrogen loss linked to anaerobic ammonium oxidation in estuarine sediments in China. *J. Soils Sediments* 17 2537–2546. 10.1007/s11368-017-1728-y

[B55] Yoshinaga I. Amano T. Yamagishi T. Okada K. Ueda S. Sako Y. (2011). Distribution and diversity of anaerobic ammonium oxidation (anammox) bacteria in the sediment of a eutrophic freshwater lake, lake kitaura, Japan. *Microbes Environ.* 26 189–197. 10.1264/jsme2.ME10184 21558678

[B56] Zhang W. Xu M. Wang B. Wang X. (2009). Soil organic carbon, total nitrogen and grain yields under long-term fertilizations in the upland red soil of southern China. *Nutr. Cycling Agroecosyst.* 84 59–69. 10.1007/s10705-008-9226-9227

[B57] Zhao Y. Xia Y. Kana T. M. Wu Y. Li X. Yan X. (2013). Seasonal variation and controlling factors of anaerobic ammonium oxidation in freshwater river sediments in the taihu lake region of China. *Chemosphere* 93 2124–2131. 10.1016/j.chemosphere.2013.07.063 23978673

[B58] Zheng Y. Jiang X. Hou L. Liu M. Lin X. Gao J. (2016). Shifts in the community structure and activity of anaerobic ammonium oxidation bacteria along an estuarine salinity gradient. *J. of Geophys. Res. Biogeosci.* 121 1632–1645. 10.1002/2015JG003300

[B59] Zhu G. Wang S. Wang Y. Wang C. Risgaardpetersen N. Jetten M. S. (2011). Anaerobic ammonia oxidation in a fertilized paddy soil. *ISME J.* 5 1905–1912. 10.1038/ismej.2011.63 21593796 PMC3223303

