# Supplementary material for: Anaerobic Ammonium Oxidation in Acidic Red Soils
Source: Front Microbiol. 2018 Sep 5;9:2142. doi: 10.3389/fmicb.2018.02142 (PMC6134040; doi:10.3389/fmicb.2018.02142)
Supplement: Supplementary file 1 [file Image_1.PDF]

Supplementary Material

Anaerobic ammonium oxidation in acidic red soils

Jiapeng Wu[1,3], Yiguo Hong[2*], Xiang He[2], Lijing Jiao[1,3], Xiaomei Wen[2], Shuai Chen[2], Guangshi Chen[2], Yiben Li[2], Tianzheng Huang[2], Yaohao Hu[2], Xiaohan Liu[2]

*** Correspondence:** Yiguo Hong: yghong@gzhu.edu.cn

1 Supplementary Figures and Tables

1.1 Supplementary Figures

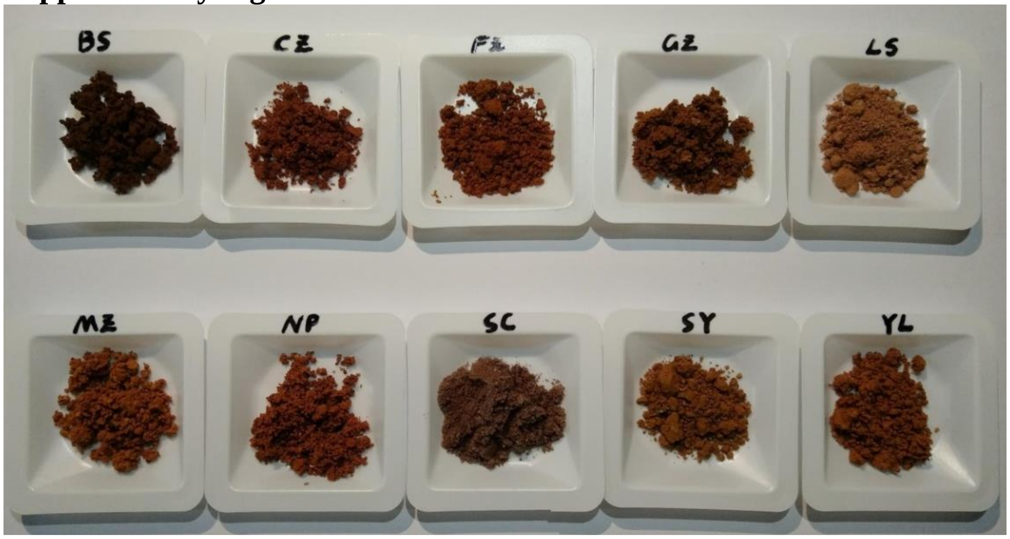

Supplementary Figure 1. Morphological characteristics of 10 red soil samples collected from 9 provinces of Southern China.

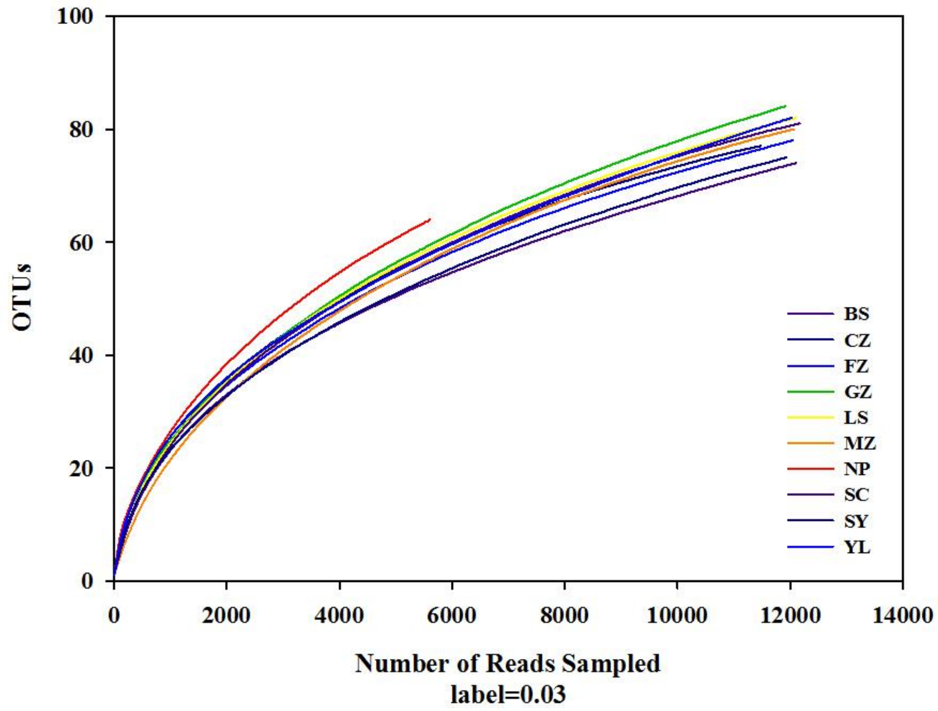

Supplementary Figure 2. Rarefaction curves for the most abundant anammox OTUs (with removed the rare OTUs) based on 97% nucleotide sequences cut offs.

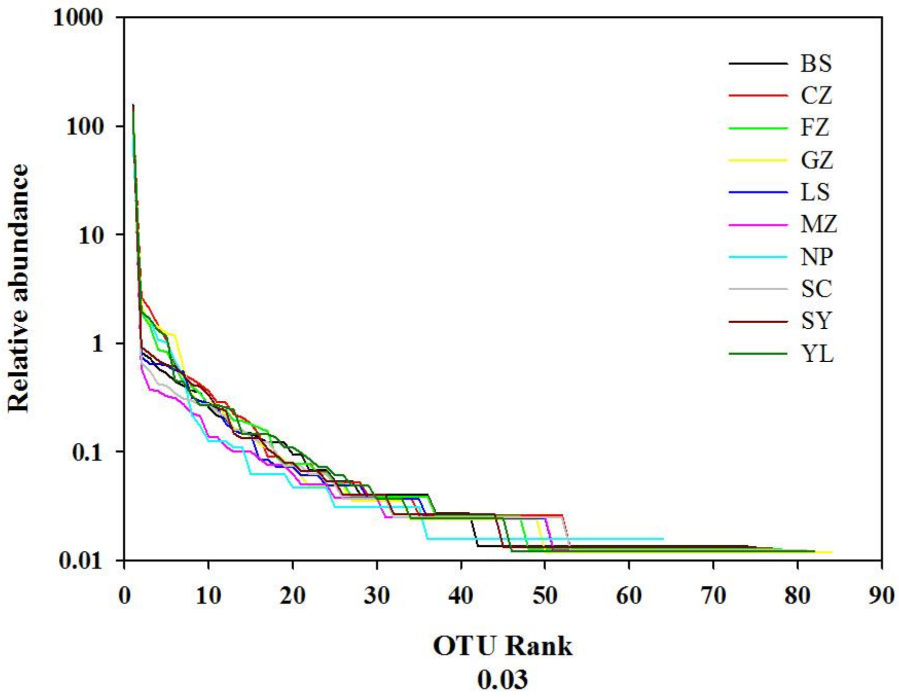

Supplementary Figure 3. The rank-abundance curves of anammox 16S rRNA gene sequences retrieved from red soils.

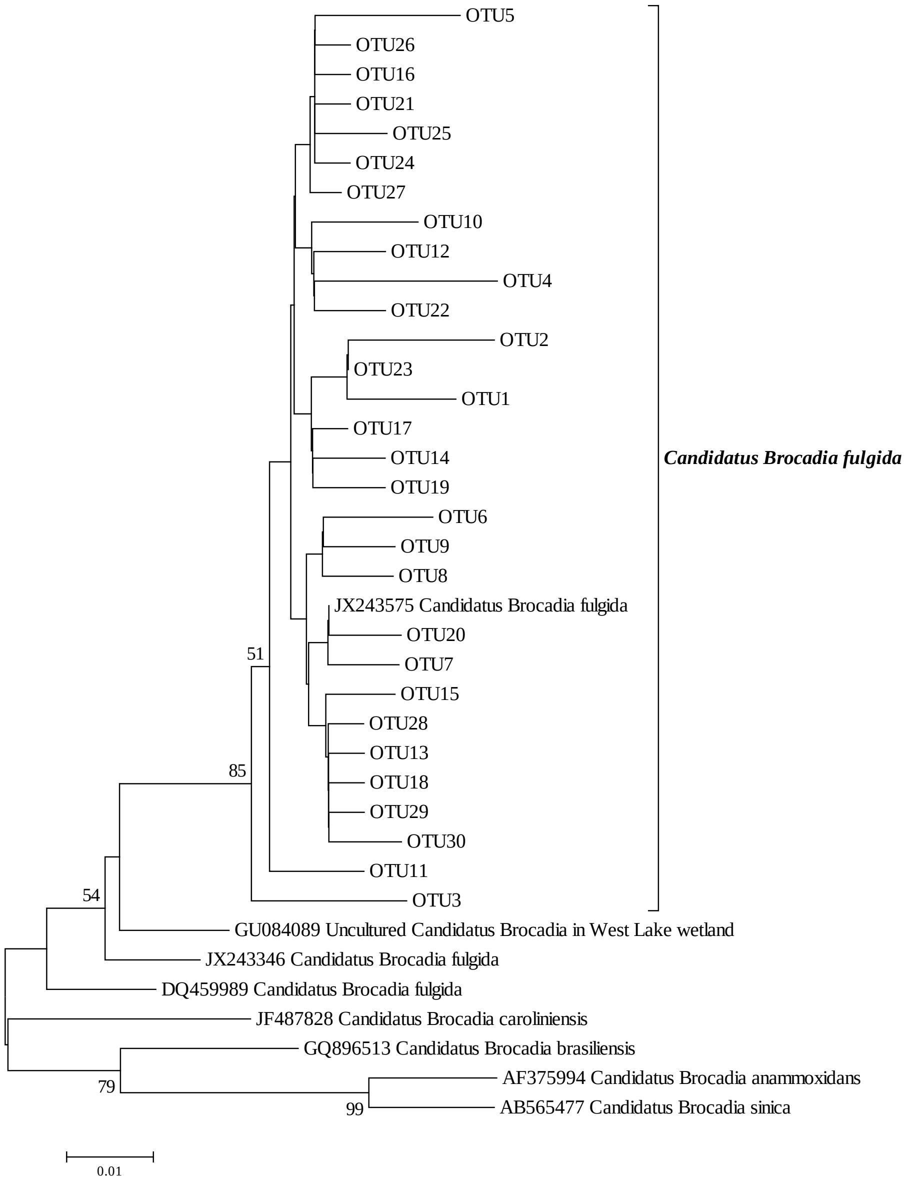

Supplementary Figure 4. Phylogenetic tree of dominant Anammox 16S rRNA OTUs in the red soils.

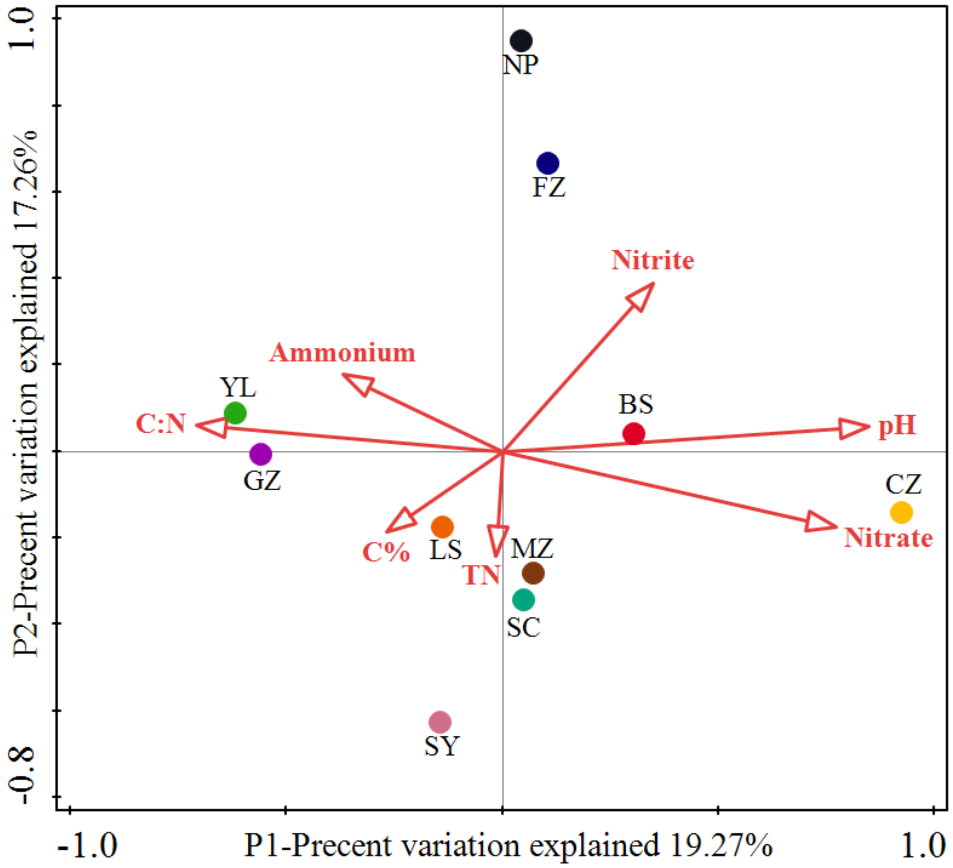

Supplementary Figure 5. Canonical correlation analysis (CCA) to measure the relationship between anammox bacterial diversity and red soil characteristics.

1.2 Supplementary Tables

Supplementary Table 1. Primer pairs in this study and correspondence PCR protocols.

Target gene	Primer pair	Sequences	PCR conditions	Methods
Anammox 16S rRNA gene	Barcode-A438f<br>A684r	5′-XXXXXXXX-GTCRGGAGTTADGAAATG-3′<br>5′- ACCAGAAGTTCCACTCTC-3′	95°C for 3 min, 35×(94°C for 45 s, 49°C for 45 s, 72°C for 45 s), 72°C for 10 min.	PCR
Anammox 16S rRNA gene	A438f<br>A684r	5′- GTCRGGAGTTADGAAATG-3′<br>5′- ACCAGAAGTTCCACTCTC-3′	95°C for 3 min,40×(95°C for 15 s, 56°C for 30 s, 72°C for 30 s).	qPCR
Anammox *hzsB gene*	HSBeta396f<br>HSBeta742r	5′- ARGGHTGGGGHAGYTGGAAG-3′<br>5′- GTYCCHACRTCATGVGTCTG-3′	95°C for 3 min,40×(95°C for 15 s, 54°C for 30s, 72°C for 30s).	qPCR

PCR was performed in a total volume of 25 µL containing GoTaq Green Master Mix (Promega）12.5 µL, each primer (15 µM) 1 µL, template DNA 1 µL and 9.5 µL of ddH_2O.

Q-PCR was performed in a total volume of 25 µL containing 12.5 µL of GoTaq qPCR Master Mix (Promega), 1 µL of each primer (15 µM), 1µL of template DNA and 12.5 µL of ddH_2O

Supplementary Table 2. Potential rates of denitrification and anammox in selected red soils.

Samples	Rate (nmol N g^{-1} dry red soil h^{-1})	
	Denitrification	Anammox
BS	1.23±0.10	0.25±0.04
CZ	1.63±0.19	0.59±0.07
FZ	0.07±0.01	0.02±0.02
GZ	0.04±0.01	0.01±0.00
LS	0.29±0.20	0.06±0.09
MZ	0.01±0.01	0.01±0.00
NP	0.02±0.02	0.02±0.02
SC	0.04±0.04	0.01±0.01
SY	0.93±0.10	0.26±0.03
YL	0.02±0.03	0.01±0.01

Supplementary Table 3. Pearson correlation analyses of anammox potential rates, gene abundances, and red soil properties

	ANA rate	*hzsB* abundance	16S rRNA abundance	pH	NH_4^+	NO_3^-	NO_2^-	TN	C%	C:N
ANA rate	1									
hzsB abundance	0.228	1								
16S rRNA abundance	0.189	0.942**	1							
pH	0.622	-0.200	-0.052	1						
NH_4^+	-0.108	-0.027	-0.017	0.103	1					
NO_3^-	0.889**	-0.133	-0.190	0.537	-0.310	1				
NO_2^-	0.443	0.320	0.179	0.232	0.111	0.209	1			
TN	0.330	0.637*	0.652*	0.241	0.588	-0.107	0.519	1		
C%	0.157	0.470	0.488	0.076	0.779**	-0.223	0.365	0.933**	1	
C:N	-0.570	-0.332	-0.355	-0.629	0.341	-0.384	-0.480	-0.300	0.047	1

*Correlation is significant at the 0.05 level.

* * Correlation is significant at the 0.01 level.
